# Place of care and death preferences among recently bereaved family members: a cross-sectional survey

**DOI:** 10.1136/spcare-2023-004697

**Published:** 2024-06-04

**Authors:** Anna O'Sullivan, Cecilia Larsdotter, Richard Sawatzky, Anette Alvariza, Henrik Imberg, Joachim Cohen, Joakim Öhlén

**Affiliations:** 1 Department of Nursing Science, Sophiahemmet University, Stockholm, Sweden; 2 Department of Health Care Sciences, Marie Cederschiold Hogskola—Campus Ersta, Stockholm, Sweden; 3 School of Nursing, Trinity Western University, Langley, British Columbia, Canada; 4 Centre for Advancing Health Outcomes, Providence Health Care, Vancouver, British Columbia, Canada; 5 Department of Research and Development, Stockholms Sjukhem, Stockholm, Sweden; 6 Statistiska Konsultgruppen, Gothenburg, Sweden; 7 Department of Molecular and Clinical Medicine, Institute of Medicine, University of Gothenburg Sahlgrenska Academy, Goteborg, Sweden; 8 End of Life Care Research Group, Vrije Universiteit Brussel, Brussels, Belgium; 9 Ghent University, Ghent, Belgium; 10 Institute of Health and Care Sciences, University of Gothenburg Sahlgrenska Academy, Gothenburg, Sweden; 11 Centre for Person-centred Care (GPCC); Palliative Centre, Sahlgrenska University Hospital, Gothenburg, Sweden

**Keywords:** Bereavement, Communication, Home Care, Hospital care, Nursing Home care, Advance Care Planning

## Abstract

**Objectives:**

The aim was: (1) to investigate preferred place for end-of-life care and death for bereaved family members who had recently lost a person with advanced illness and (2) to investigate associations between bereaved family members’ preferences and individual characteristics, health-related quality of life, as well as associations with their perception of the quality of care that the ill person had received, the ill person’s preferred place of death and involvement in decision-making about care.

**Methods:**

A cross-sectional survey with bereaved family members, employing descriptive statistics and multinominal logistic regression analyses.

**Results:**

Of the 485 participants, 70.7% were women, 36.1% were ≥70 years old, 34.5% were partners and 51.8% were children of the deceased. Of the bereaved family members, 52% preferred home for place of end-of-life care and 43% for place of death. A higher likelihood of preferring inpatient palliative care was associated with being female and having higher education, whereas a lower likelihood of preferring a nursing home for the place of care and death was associated with higher secondary or higher education. Partners were more likely to prefer hospital for place of care and nursing home for place of death.

**Conclusions:**

Home was the most preferred place for end-of-life care and death. Bereaved people’s experiences of end-of-life care may impact their preferences, especially if they had a close relationship, such as a partner who had a higher preference for nursing home and hospital care. Conversations about preferences for the place of care and death considering previous experience are encouraged.

**WHAT IS ALREADY KNOWN ON THIS TOPIC**

- Home is often described as the ideal place for end-of-life care and death. To be cared for and die in one’s preferred place can be considered an essential part of person-centred care at the end of life. Previous studies focusing on the general public and people with advanced illness have shown that the preferred place for most is at home. Little is known about preferences of bereaved people regarding the place of end-of-life care and death and the impact of their previous experiences as family members.

**WHAT THIS STUDY ADDS**

- The majority of the bereaved family members in this study preferred place for end-of-life care and death at home. Bereaved people’s experiences of end-of-life care may impact their preferences, especially when involving a close relationship such as a partner who had a higher preference for nursing home and hospital care.

**HOW THIS STUDY MIGHT AFFECT RESEARCH, PRACTICE OR POLICY**

- Considering previous experiences of end-of-life care and death in inquiries about preferences is important, as is taking into account who is responding—general population, severely ill people or people with experience regarding end-of-life care and death. This is vital to consider in future research and for advance care planning, as well as for policy-making to organise care based on research and preferences. In order to develop policy and appropriate care according to needs of the total population in a country, knowledge of such preferences among different groups within the population is needed.

## Background

 International studies show that most people prefer to receive care and die in their own home, assuming that high-quality care can be provided.^1^,^3^ Place of death and end-of-life care has become an important public health issue, as a result of demographic and epidemiological changes resulting in longevity with chronic illness.^4^

To die in one’s preferred place is internationally considered a quality indicator of end-of-life care.^5^ Where people die, however, varies largely between countries and different patient groups.^6 7^ A study in 14 countries investigating place of death for people with potential palliative care needs to found a variation between 13% and 53% in deaths occurring at home. Deaths in hospital span between 25% and 85% and deaths in long-term facilities (ie, nursing homes) varied between 1% and 35%,^6^ with the majority of people in high-income countries still dying in institutions.^7^ In Sweden, one in five die in their own home, while most die in hospitals and nursing homes.^8 9^ In a study in the UK about preferred and actual place of death in patients with haematological cancer, almost half had discussed their preferred place of death. Of those, around half wished to die at home, a third in hospital and almost a fifth in a hospice, with 6 in 10 dying in their preferred place.^10^ An American study on patients with advanced cancer cared for in a specialised palliative care unit or at home with outpatient specialised palliative home care showed that 58% of the patients in specialised palliative care units preferred their home for the place of death, as did 72% of the patients with outpatient care.^11^

Studies about preferences for place of end-of-life care and death in populations following a recent bereavement of a close person (ie, partners, parents, children, siblings, friends, neighbours and others) are sparse. Preferences for place of end-of-life care and death may vary considering infrastructure, culture and illness. In a report from the UK, only 35% of deceased individuals had stated a preferred place of death, most of whom preferred to die at home.^12^ A Japanese study of preferences for place of end-of-life care and death in the general population showed variations depending on a hypothetical illness situation, for cancer 50% preferred home; for end-stage heart disease, 31% preferred home and 51% a medical facility; and for dementia, 55% preferred nursing home and 30% a medical facility.^13^ A study in a general Swedish population showed home as the preferred place for both the end-of-life care (68%) and death (71%).^14^

Previous research shows that the place for care at the end of life can be of great importance to both the deceased person and family members.^15^ That is, family members are often central to—and take great responsibility for—the care of people with advanced illness, which has been shown to be both rewarding and burdensome.^16 17^ Severe depressive symptoms have been shown to be associated with decreased health-related quality of life (HRQoL) among bereaved family members, linked to caregiver burden^18^—and decreased HRQoL ratings have been reported after the loss of a family member.^19 20^ Hence, it is important to observe HRQoL in bereaved family members as well as understand their preferences for care setting.

Bereaved family members’ previous experiences and satisfaction with care that the person with advanced illness have received in different care places may have an impact on their own preferences for end-of-life care and death. In order to develop policy and care according to the needs of a country’s total population, knowledge of preferences regarding end-of-life care and death among different groups within the population is needed. Hence, the aim of this study was (1) to investigate preferred place for end-of-life care and death for bereaved family members who had recently lost a person with advanced illness and (2) to investigate associations between bereaved family members’ preferences and individual characteristics, HRQoL, as well as associations with their perception of the quality of care that the ill person had received, the ill person’s preferred place of death and involvement in decision-making about care.

## Methods

### Study design

A retrospective cross-sectional survey.

### Sample and setting

The study sample included 1277 adults who had recently lost a person with advanced illness at one of four hospitals in two healthcare regions in Sweden (August 2016–April 2017). In this study, the term family members refer to spouses/partners, children, siblings, parents, friends and other people listed as the persons closest to the deceased persons. Hospital is the most common place of death in Sweden, and our choice of regions (Stockholm and the Southeast healthcare region) was based on results from a previous population-based place of death study that indicated that, in the Stockholm region, hospital deaths were significantly more likely than in other regions, while home deaths were more likely in the Southeast healthcare region compared with other regions.^9^

The sample was a total population sample, including all the deceased persons in the study settings during the inclusion period who fulfilled the inclusion criteria. Criteria for inclusion were: an identifiable bereaved family member of the deceased person; ≥18 years old (both deceased persons and bereaved family members); underlying causes of death (International Classification of Diseases and Health Related Problems—10 codes) in accordance with the Murtagh *et al*^21^ model (diagnoses with potential palliative care needs); and time of death no less than four and no more than 12 months before study recruitment.

In Sweden, end-of-life care can be provided at home, in hospitals, in nursing homes and in specialised palliative care units, for example, inpatient palliative care units or hospices. The deceased persons had all died in hospital but had received care in several care places and settings.

### Recruitment and data collection

Of all the patients who died in the recruitment hospitals during the study period, 78% (n=1277) were eligible for inclusion. Hospital administrators identified the deceased persons and one healthcare professional at each hospital identified the bereaved family members who were listed in the hospital’s patient records as the closest person to the patient. Postal addresses of the bereaved family members were retrieved from publicly available databases. Only one family member per deceased person was invited to the study. The survey was sent via post to the bereaved family members 4–12 months after the death, including contact information for one of the researchers (author 1) and information stating that the study was performed in cooperation with the hospital in which their family member had died. With the questionnaire, the participants received written information about the study and were informed that the data would be completely confidential and that they could withdraw from the study at any time without any explanation. A returned questionnaire was considered as consent; no other written informed consent for participation in the study was provided. All collected data have been carefully handled to ensure confidentiality. Data containing personal information have been coded and anonymised, and thereafter only used in coded form. The key to the coding and the identification of the participants were destroyed. The data have only been accessed by members of the research group.

### Study variables

Three questions about the place of care and death that respondents would most and least prefer in case of serious illness, and what aspects of care would be the most important, were used—derived from the PRISMA study. PRISMA is a European Commission project funded by the seventh framework programme called ‘Reflecting the Positive diveRsities of European prIorities for reSearch and Measurement in end of life cAre’ (PRISMA), a collaborative effort with the aim to coordinate high-quality research into end-of-life cancer care^22^ (table 1). Additionally, the questionnaire included the instrument Views of Informal Carers—Evaluation of Services—short form (VOICES (SF)), initially developed in the UK.^23^ Both these surveys were translated from English into Swedish and validated in 35 bereaved family members.^24^ The following items from the VOICES (SF) were used for this study: overall care quality for all care during the last 3 months added together, support family members received from health and social services during the illness period, the deceased person’s preferred place of death, whether the deceased person had died in an appropriate place and the ill person’s as well as the family member’s involvement in decision-making. All these items are only based on the bereaved family members’ reports, considered as proxies with regard to questions about the deceased persons’ involvement and preferences. To assess the participants’ HRQoL, we used RAND-12, which is included in the survey based on the Swedish version of RAND-36.^25^ RAND-12 is a short version of the RAND-36 commonly used to evaluate HRQoL. It contains 12 items on a 3–6 point Likert scale and has two composite scores—Physical Composite Score (PCS) and Mental Health Composite Score (MCS).^26^

**Table 1:** Questions concerning preferences for place of care and death

Question	Responses
1. In a situation of serious illness, such as cancer, with less than a year to live, where do you think you would prefer to be cared for if the circumstances (assuming you get the care and support you need) made it possible for you to choose?	In your own home. In the home of a relative or a friend. In a hospice or palliative care unit—places with specialised care and beds for dying patients. In hospital—but not in a palliative care unit. In a nursing home. Somewhere else.
2. In a situation of serious illness, such as cancer, with less than a year to live, where do you think you would prefer to die if the circumstances (assuming you get the care and support you need) made it possible for you to choose?	In your own home. In the home of a relative or a friend. In a hospice or palliative care unit—places with specialised care and beds for dying patients. In hospital—but not in a palliative care unit. In a nursing home. Somewhere else.
3. What would be most important to you in the care that is available?	Having as much information as you want. Choosing who makes decisions about your care. Dying in the place you want.

Additionally, the survey included background questions about the participants and the deceased persons (sex, age, time of illness before death and diagnosis—cancer/non-cancer). The participants’ sex, age, educational level, occupation, relationship to the deceased person and country of birth, as well as country of birth of the participant’s parents, were used as independent variables.

### Statistical analyses

Initially, descriptive statistics were performed, and the PCS and MCS sum scores for RAND-12 were analysed according to Ware *et al*.^27^ Analyses of preferences for care and death in relation to both the family members’ and the deceased persons’ characteristics were performed with multinomial logistic regression.

Statistical analyses were performed using univariable and multivariable multinomial logistic regression. Multivariate likelihood ratio tests were used to evaluate the associations with the preferred place of care and death. Models for multivariable analyses were developed using a two-stage procedure as follows. First, all variables significant at the 25% significance level in univariable analyses were selected. Among these, a final multivariable model was selected using the Least Absolute Shrinkage and Selection Operator (LASSO).^28^ The LASSO penalty was chosen based on Akaike’s Information Criterion (AIC), and the model with the lowest AIC was selected. ORs from the univariable and final multivariable models are presented along with 95% Wald CIs. The rationale for using LASSO was that conventional stepwise approaches are at increased risk of leading to variable selection bias, which is increasingly regarded as a serious problem in health research.^29^

Missing data were handled by multiple imputation using fully conditional specification with 50 imputed datasets.^30 31^ This assumes data to be missing at random and that missingness is ignorable when accounting for an appropriate set of covariates that explain the missing data mechanism. As auxiliary variables for the imputation, we included all variables that at the 20% significance level were related to the variable to be imputed or related to missingness in that variable. Each imputed observation was weighted by the reciprocal of the number of imputations, so that observations with complete data and missing data all contributed with a weight of one.

All tests were two sided and conducted at the 5% significance level. Statistical analyses were performed using IBM SPSS Statistics, V.27.0 and using SAS/STAT Software, V.9.3 of the SAS System for Windows were used. The MI procedure was used for multiple imputation, the MIANALYZE procedure for pooling results across imputations and the HPGENSELECT procedure for model selection using LASSO.

## Results

The response rate was 37.9%, resulting in a total of 485 bereaved family members participating in the study.

### Characteristics of the family members and the deceased persons

The family members were between 18 and ≥90 years, 70.7% were women, 29.5% had a lower secondary education, 30.5% had a higher secondary education and 39.4% had a higher education. About half (51.8%) were children of the deceased person and 34.5% were spouses or partners. Of all the participants, 92.1% were born in Sweden and 52.4% were retired. RAND-12 MCS had a mean score of 47.8 (SD: 12.1), and the mean score for PCS was 48.1 (SD: 10.4) (table 2). The non-responders’ individual characteristics were not available. There were, however, no differences for the deceased individuals linked to the non-responding family members regarding the available characteristics, which were age, sex and diagnosis, compared with the deceased individuals linked to the responding family members.

**Table 2:** Characteristics of the family members

Variables	N	Per cent*
Sex (missing=0)†		
Male	142	29.3
Female	343	70.7
Age (years) (missing=8)†		
18–39	12	2.4
40–59	141	29.1
60–69	52	31.3
70–79	111	22.9
80–89	55	11.3
90+	6	1.2
Educational attainment (missing=3)†		
Lower secondary education	143	29.5
Higher secondary education	148	30.5
Higher education	191	39.4
Occupation (missing=12)†		
Employed/self-employed/student/home maker	211	44.6
Pensioner	249	52.6
Unemployed/long-term sick/other	13	2.7
Country of birth (missing=1)†		
Sweden	446	92.1
Other country	38	7.9
Family member’s parents’ country of birth (missing=3)†		
Sweden	419	86.9
Other country	63	13.1
RAND-12 MCS (missing=56)†	429	Mean: 47.8 (SD: 12.1)Median: 51.8 (min: 12.7–max: 70.6)
RAND-12 PCS (Missing=56)†	429	Mean: 48.1 (SD: 10.4)Median: 52.0 (min: 16.2–max: 64.3)
Relationship to the deceased person (missing=4)†		
Spouse	166	34.5
Child	249	51.8
Other‡	66	13.7

* Column percentage displayed.

† Missing = (x) shows number of missing cases.

‡ For example,E.g., parent, sibling, friend.

MCSMental Health Composite ScorePCSPhysical Composite Score

The deceased persons were between 40 and ≥90 years (64% were 80 or older), 50.3% were men, 70.3% died from diseases other than cancer and 43.0% had been ill for a year or longer before death. Of the deceased persons, 79.2% had been cared for at home at some point during the last 3 months of life; 27.4% in nursing home care; and 15.7% in a specialised palliative care unit.

Of the family members, 79% rated the overall care of their deceased relative (all care summarised) during the last 3 months as high. Of the family members, more than half (61%) reported that they received as much help and support as they wanted during the illness period of the deceased person. However, about a third reported that they did not. Family members reported that 60% of the ill persons had been involved as much as wanted in decision-making about their own care, and 66% of the family members were involved as much as they had wanted in the ill person’s care. A majority (70%) of the deceased persons had not expressed where they wanted to die. Of the 122 who had expressed where they wished to die, 50% had said at home and 22% in the hospital. All the deceased persons included in the study had died in hospital because the sample was drawn from hospital deaths and 81% of the family members reported that the ill person had died in the right place (table 3).

**Table 3:** Descriptive results include the overall quality of care, the involvement of the ill person and family members in decision-making and the ill person’s preferred place of death

Variables	Participants(n 485)*
Quality of care during last 3 months	
Outstanding	38 (8.0%)
Excellent	151 (31.8%)
Good	185 (38.9%)
Fair	61 (12.8%)
Poor	25 (5.3%)
Unknown	15 (3.2%)
Missing†	10
Support for family members	
Yes, we got as much support as we wanted	285 (61.4%)
Yes, we got some support, but not as much as we wanted	91 (19.6%)
No, although we tried to get more help	27 (5.8%)
No, but we did not ask for more help	28 (6.0%)
We did not need any help	33 (7.1%)
Missing	21
Ill person involvement in decision-making	
As much as wanted	282 (59.5%)
Wanted to be more involved	51 (10.8%)
Wanted to be less involved	5 (1.1%)
Unknown	136 (28.7%)
Missing	11
Family involvement in decision-making	
As much as wanted	309 (65.1%)
Wanted to be more involved	114 (24.0%)
Wanted to be less involved	3 (0.6%)
Unknown	49 (10.3%)
Missing	10
Did he/she ever say where he/she wanted to die?	
Yes	94 (20.0)
No	331 (70.3)
Unsure	46 (9.7)
Missing	14
Ill person’s preferred place of death	
At home	61 (50.0%)
Inpatient palliative care unit	2 (1.6%)
Hospital	27 (22.1%)
Nursing home	3 (2.5%)
Did not matter	21 (17.2%)
Changed his/her mind	1 (0.8%)
Other	7 (5.7%)
Not stated or missing	363
Died in the right place	
Yes	390 (81.3%)
No	46 (9.6%)
Unsure	44 (9.2%)
Missing	5

* Column percentage displayed.

† Missing values are excluded from the analyses.

### Bereaved family members’ preferences for their own place of care and death

**Figure 1:**
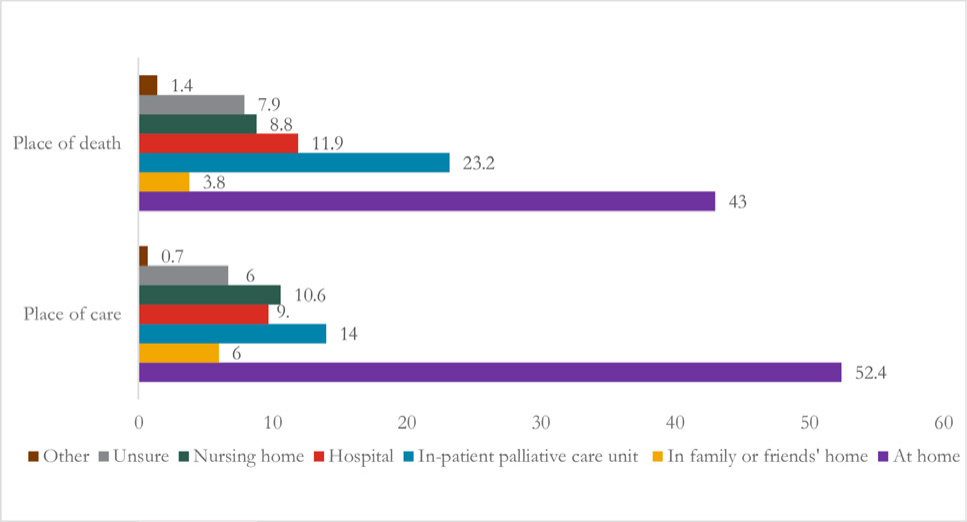
Percentage of bereaved family members’ preferences for place of end-of-life care and death. Missing cases (n=50 for place of care and n=41 for place of death) excluded from the analyses.

Of all the participating bereaved family members, 52% reported preferring to be cared for at home in a situation of serious illness, such as cancer, with less than a year to live, assuming care and support were available. Another 14% preferred to be cared for in an inpatient palliative care unit, followed by nursing home and hospital care (figure 1). The most preferred place of death in a situation of serious illness, such as cancer with less than a year to live, assuming care and support were available, was home (43%), followed by an inpatient palliative care unit or hospice (23%), a hospital (12%) and a nursing home (9%) (figure 1).

For the family members, the most important aspects regarding care, in case of a serious illness scenario, were receiving as much information as wanted (47%), to die in one’s preferred place (29%) and being able to choose who makes decisions about the care (18%).

### Associations between bereaved family members’ preferred place of their own end-of-life care and individual characteristics, HRQoL and perceived care quality

In univariable analyses, there was a higher likelihood of preference for end-of-life care at hospice, compared with being cared for at home, among family members of older age (p=0.048), female (p=0.002) and among partners (vs children) to the deceased person (online supplemental table 1). Similarly, there was a stronger preference for care in a nursing home if the family member was older (p=0.007), with lower education (p=0.002) and a partner to the deceased person (p=0.0004) (online supplemental table 1). In multivariable analyses, there were associations between family members’ individual characteristics and their preferred place of care, with a higher likelihood to prefer inpatient palliative care unit if female (OR: 4.07; 95% CI: 1.66 to 9.96) and in the case of having a higher secondary education (OR: 3.17; 95% CI: 1.25 to 8.06) or a higher education (OR: 2.73; 95% CI: 1.15 to 6.46). There was a lower likelihood of preferring nursing home for place of care if the family member had a higher secondary (OR: 0.37; 95% CI: 0.15 to 0.94) or a higher education (OR: 0.22; 95% CI: 0.09 to 0.54). Being a partner to the deceased person was associated with a higher likelihood of preferring hospital for place of care (OR: 2.98; 95% CI: 1.14 to 7.79) (table 4). No significant associations were found between family members’ preferred place of care and their HRQoL, their perceived quality of the deceased person’s end-of-life care nor for if the deceased person had died in the right place according to his/her family member or for involvement in decision-making about care (online supplemental table 1).

**Table 4:** Where would you most like to be cared for?

Variables	Comparison	Family or friends’ homeOR (CI)	HospiceOR (CI)	HospitalOR (CI)	Nursing homeOR (CI)
Deceased person’s sex	Female versus male	0.94 (0.40 to 2.23)	1.00 (0.50 to 1.97)	0.60 (0.29 to 1.26)	0.73 (0.33 to 1.64)
Age	≤59 vs 60–69	2.71 (0.75 to 9.70)	0.81 (0.33 to 1.97)	0.57 (0.21 to 1.56)	0.59 (0.20 to 1.75)
	70+ vs 60–69	0.79 (0.21 to 2.96)	2.22 (0.81 to 6.11)	0.47 (0.19 to 1.19)	0.90 (0.35 to 2.31)
Sex	Female versus male	1.13 (0.45 to 2.86)	4.07 (1.66 to 9.96)	1.13 (0.53 to 2.42)	1.19 (0.52 to 2.74)
Educational level	Higher secondary versus elementary secondary	0.95 (0.30 to 3.03)	3.17 (1.25 to 8.06)	1.22 (0.48 to 3.14)	0.37 (0.15 to 0.94)
	Higher versus elementary secondary	0.72 (0.23 to 2.21)	2.73 (1.15 to 6.46)	1.17 (0.51 to 2.69)	0.22 (0.09 to 0.54)
Occupation	Employed/student* versus unemployed/pensioner†	0.45 (0.12 to 1.67)	1.23 (0.45 to 3.38)	0.87 (0.32 to 2.35)	1.65 (0.59 to 4.58)
Relation to deceased person	Partner versus child	0.93 (0.26 to 3.38)	1.93 (0.75 to 4.97)	2.98 (1.14 to 7.79)	2.03 (0.78 to 5.24)
	Other versus child	1.21 (0.31 to 4.81)	1.55 (0.63 to 3.83)	2.16 (0.76 to 6.17)	1.66 (0.53 to 5.16)
RAND-12 Physical Composite Score per 10 units		0.86 (0.57 to 1.31)	1.08 (0.77 to 1.52)	0.76 (0.55 to 1.07)	0.94 (0.67 to 1.31)

Statistical analyses were performed using multivariable multinomial logistic regression. All variables significant at the 25% significance level in univariable analyses and subsequently selected by Least Absolute Shrinkage and Selection Operator (LASSO) are included in the analyses. The following study variables were excluded by model selection: Ddeceased person’s age; Ffamily members’ country of birth; Ffamily members’ parents’ country of birth; Ffamily members’ occupation; Ttime of illness before death, Ccancer/non-cancer; RAND-12 MCSMental Health Composite Score; Qquality of care during last 3 months; Ssupport for family members; Iill person participation in decision-making; Ffamily participation in decision-making; Iill person’s preferred place of death; Ddeath in the right place.

Results from the multivariable multinomial logistic regression analysis, selected by LASSO. OR (95% CI) versus at home.

* Employed/student/self-employed/home maker/student.

† Unemployed/pensioner/long-term sick/other.

### Associations between bereaved family members’ preferred place of their own death and individual characteristics, HRQoL and perceived care quality

In the univariable analyses, increased preferences for dying in a hospice (vs home) were found associated with family members of older age (p=0.032) and female (p=0.003), as well as among partners (vs children) to the deceased person (p=0.038). The preferences for dying in a nursing home also increased with family members age (p=0.0006) and lower education (p=0.003) of the family member, as well as among unemployed family members (p=0.002) and partners to the deceased person (p<0.0001) (online supplemental table 2). In multivariable analyses, there were also associations between the family members’ individual characteristics and their preferred place of death: was a higher likelihood of preferring inpatient palliative care unit among females (OR: 2.30; 95% CI: 1.22 to 4.32) and among those with a higher education (OR: 2.43; 95% CI: 1.17 to 5.03). There was a lower likelihood of preferring nursing home for place of death if family members had a higher education (OR: 0.34; 95% CI: 0.13 to 0.92), and higher likelihood of preferring nursing home for place of death if the relationship to the deceased person was partner (OR: 5.02; 95% CI: 1.72 to 14.61) or other (OR: 3.65; 95% CI: 1.13 to 11.76) (table 5). No significant associations were found between family members’ preferred place of death and their HRQoL, their perceived quality of the deceased person’s end-of-life care nor for if the deceased person had died in the right place according to his/her family member or for involvement in decision-making about care (online supplemental table 2).

**Table 5:** Where would you most like to die?

Variables	Comparison	Family or friend’s home	Hospice	Hospital	Nursing home
Deceased person’s sex	Female versus male	1.50 (0.50 to 4.48)	0.81 (0.47 to 1.38)	0.80 (0.41 to 1.59)	0.77 (0.33 to 1.80)
Age	≤59 vs 60–69	3.21 (0.59 to 17.38)	1.17 (0.57 to 2.38)	1.04 (0.41 to 2.63)	0.86 (0.21 to 3.48)
	70+ vs 60–69	0.63 (0.14 to 2.73)	1.97 (0.84 to 4.62)	0.70 (0.28 to 1.78)	0.61 (0.22 to 1.69)
Sex	Female versus male	0.52 (0.17 to 1.60)	2.30 (1.22 to 4.32)	1.16 (0.57 to 2.36)	0.99 (0.40 to 2.45)
Educational level	Higher secondary versus elementary secondary	0.36 (0.08 to 1.69)	1.75 (0.79 to 3.89)	1.49 (0.62 to 3.59)	0.57 (0.21 to 1.57)
	Higher versus elementary secondary	0.73 (0.21 to 2.62)	2.43 (1.17 to 5.03)	1.37 (0.59 to 3.15)	0.34 (0.13 to 0.92)
Occupation	Employed/student* versus unemployed/pensioner†	0.36 (0.07 to 1.75)	0.97 (0.44 to 2.14)	0.52 (0.21 to 1.29)	0.73 (0.20 to 2.62)
Death in the right place	Yes versus no	0.37 (0.10 to 1.35)	1.43 (0.60 to 3.40)	4.24 (0.92 to 19.57)	Non-estimable
Relation to deceased person	Partner versus child	2.64 (0.62 to 11.16)	1.34 (0.61 to 2.94)	2.02 (0.80 to 5.08)	5.02 (1.72 to 14.61)
	Other versus child	1.80 (0.34 to 9.51)	1.14 (0.53 to 2.46)	0.64 (0.20 to 2.10)	3.65 (1.13 to 11.76)

Statistical analyses were performed using multivariable multinomial logistic regression. All variables significant at the 25% significance level in univariable analyses and subsequently selected by the Least Absolute Shrinkage and Selection Operator (LASSO) are included in the analyses. The following study variables were excluded by model selection: Ddeceased person’s age; Ffamily member’s country of birth; Ffamily member’s parents’ country of birth; Ttime of illness before death, Ccancer/non-cancer; RAND-12 PCSPhysical Composite Score; RAND-12 MCSMental Health Composite Score; Qquality of care during last 3 months; Ssupport for family members; Iill person participation in decision-making; Ffamily participation in decision-making; Iill person’s preferred place of death.

Results from the multivariable multinomial logistic regression analysis, selected by LASSO. OR (95% CI) versus at home.

* Employed/student/self-employed/home maker/student.

† Unemployed/pensioner/long-term sick/other.

## Discussion

Our study indicated that most bereaved family members named home as their preferred place for end-of-life care and death, and this was preferences was associated with the bereaved family members’ own individual characteristics (sex, educational attainment and the relationship to the deceased person). Other variables such as HRQoL, quality of care their deceased person had received, the deceased persons’ preferred place of death and involvement in decision-making about care were not associated.

This study’s result, with home as the most preferred place for end-of-life care as well as place of death, is in line with previous studies in the general public.^14 32^ The proportion preferring home for place of death was, however, lower in this study and the percentage preferring hospital and nursing home was more than double that found in a study in Swedish general population.^14^ The preference for home as the place for end-of-life care and death in the present study is slightly lower than in a study in Japanese bereaved people, in which about two-thirds preferred home for place of end-of-life care and for death. The percentage preferring the hospital was half that of this study and about a third preferred a palliative care unit, which is substantially more than in the present study.^33^ The bereaved family members’ previous experiences of the ill person’s end-of-life care in nursing homes and hospitals and death in hospital may have influenced their own preferences for care and place of death in these place and resulting in a lower proportion preferring home in the present study.

In this study, the odds of preferring nursing home for place of death and hospital for place of care were greater if they were a partner of the deceased person. Previous research suggests that partners are more involved and informed about the care of an ill person.^34^ Greater insight of partners into the care and illness trajectory—including seeing the stress and pressure of managing at home both as a partner and as a patient—may affect this preference. Additionally, the loss of a partner means no longer having a partner providing informal care and dying at home might mean dying alone and people with children may not want to impose informal care on their children, which might explain a lesser preference for home. Furthermore, death literacy—the knowledge and understanding of end-of-life and death care options—has previously been shown to be positively influenced by being widowed and lived experiences of end-of-life care and death, both informal and formal through professional or volunteer work.^35^ The bereaved family members’ preferences for place of care at the end of life, place of death and what is most important in end-of-life care are most likely influenced by their increased death literacy through their experiences as family members of a severely ill and dying person.

Only a quarter of the family members in the present study reported that the deceased person had expressed a preference for place of death and of those, about half preferred home and almost a quarter hospital. This is slightly less than in previous research, in which about 40% of the patients had had a conversation about preferred place of death according to their medical records.^10^ This needs to be further investigated but indicates that a conversation about preference for place of death did not occur and that communication could be improved, which is important to consider for future estimates about preferences for place of end-of-life care and death. Previous studies have shown barriers for efficient communication between family and patients, for example, differing views on preferences regarding disclosure about prognosis, difficulties in approaching conversations about end-of-life issues due to not being prepared to have such conversations and not having opportunities to discuss.^36^ One study suggests that deaths of persons close may serve as both positive and negative role models that influence one’s own end-of-life preparations and that practitioners should encourage patients to use conversations about others’ deaths as springboards for discussions about one’s own end-of-life care and to engage in advance care planning together with family.^37^ Furthermore, it is important to consider who responds—general population, severely ill people or people with experiences from end-of-life care and death. This is all important to consider in future research and for advance care planning, as well as for policy-making to organise care based on research and preferences.

## Study limitations

The sample was of a population of bereaved family members of persons who had all died in hospital. The reasoning behind this sample strategy was that people who die in hospital also have experiences from other types of care. However, selecting bereaved family members of persons who died in hospital may have impacted the results. A sample of bereaved family members of persons who had all died at home would have probably had different experiences, which may have impacted their preferences. In Sweden, there is, however, barely a fifth of the population that die at home, and of those persons with cancer are more prominent, which also may impact preferences. Of the participants, 70% were women who may appear disproportionate. However, in Sweden and other countries, it is more common for women than men to provide informal care.^38 39^ The choice to group the sample’s occupations unemployed, long-term sick and other into one was justified to enable statistical analysis. We do, however, realise that there may be meaningful differences between these groups. For the analyses the categories parent, sibling, friend and others were merged into the relationship category ‘other’ to enable analysis. We realise they may differ and have different obligations, which could be of importance. A limitation is that the sample consisted mainly of native Swedes, which is not representative of pluralism in Sweden. This may have influenced the reported preferences for place of end-of-life care and death, given that traditions and views on informal care, care at the end of life and death varies between different cultures and countries, which may also impact preferences. Another limitation is that we do unfortunately not have any information about the deceased persons’ and the bereaved family members’ living situation. In Sweden, cohabiting as a partner is considered equivalent to being married. The sample consisted of family members who had recently lost a person that died in one of four hospitals in two Swedish healthcare regions; hence, the results of this study are not to be considered generalisable on a population level. However, this study provides new and important knowledge about bereaved people’s preferred place for end-of-life care and death, and possible associations between preferences, individual characteristics, HRQoL and reported quality of care that the deceased person had received, the deceased person’s preferred place of death and involvement in decision-making about care.

The response rate was rather low (37.9%) and could potentially have been improved by reminders. It was, however, an ethical choice not to distress the bereaved family members. The response rate is in line with—or higher—than similar survey studies.^40 41^ About 11% of the responses for questions about preferences for end-of-life care and place of death were missing, due to participants responding several alternatives or not at all, potentially indicating that the questions may be difficult to understand or answer. How the questions of preferences for place of end-of-life and death are posed is important, and the same can be said of the response options; the option ‘it does not matter’ or ‘it depends’ are seldom available.^42^ In the current study, these response options were not available, which is important to consider for future studies. Others’ knowledge about patient preferences is not guaranteed, likewise the reports of death in the right place are to be viewed with caution, since it is according to the bereaved person. The varying numbers for the questions about if the ill person had stated a preference, actual preference expressed and whether the person died in the right place, reflect the bereaved persons’ perspectives, which may not be the same as those of the dying person. A lack of concordance between ill persons’ and bereaved family members’ reports about care has been suggested.^43^

## Conclusions

In this study, the most preferred place for end-of-life care and death was at home. Bereaved people’s previous experiences of end-of-life care may influence their preferences, especially if they had a close relationship to the person who died, such as a partner who had a higher preference for nursing home and hospital care. For the future, this needs to be considered in research and for the organisation of end-of-life care. It is important to consider previous experiences of care and death in inquiries about preferences regarding place of end-of-life care and death, and to investigate people’s preferences on a larger scale, to inform the organisation of care and further enable the fulfilment of preferences. Furthermore, the possibility for and availability of end-of-life care and death at home needs to be extended to meet people’s preferences.

## supplementary material

10.1136/spcare-2023-004697 online supplemental file 1

## Data Availability

Data are available on reasonable request.
