# Supplementary material for: Place of care and death preferences among recently bereaved family members: a cross-sectional survey
Source: BMJ Support Palliat Care. 2024 Jun 4;14(e3):e004697. doi: 10.1136/spcare-2023-004697 (PMC11672062; doi:10.1136/spcare-2023-004697)
Supplement: online supplemental file 1 [file spcare-14-e3-s001.pdf]

Supplemental material - Preferences for place of care and death among recently bereaved family members: a cross-sectional survey study

Supplementary table 1. Where would you most like to be cared for? Results from univariable multinomial logistic regression analysis. Odds Ratio (95% CI) vs. At home.

		Odds Ratio (95% CI) vs at home				
Variable	Comparison	Family or friend's home	Hospice	Hospital	Nursing home	Global test of association†
Deceased person's age per 10 years		1.06 (0.71; 1.59) p=0.76	0.93 (0.72; 1.20) p=0.56	0.81 (0.61; 1.07) p=0.14	1.04 (0.78; 1.40) p=0.78	p=0.56
Deceased person's sex	Female vs. Male	0.92 (0.41; 2.05) p=0.84	0.61 (0.35; 1.06) p=0.081	0.53 (0.28; 1.00) p=0.049	0.57 (0.31; 1.06) p=0.076	p=0.14
Cause of death	Cancer vs. Non-cancer	0.67 (0.27; 1.63) p=0.37	0.85 (0.46; 1.58) p=0.62	0.67 (0.32; 1.41) p=0.29	1.03 (0.55; 1.94) p=0.93	p=0.75
Time of illness before death*	<24 h vs. >1 year	1.61 (0.47; 5.45) p=0.45	1.24 (0.42; 3.64) p=0.69	0.87 (0.24; 3.08) p=0.82	0.55 (0.12; 2.53) p=0.45	p=0.41
	24 h - 1 week vs. >1 year	1.97 (0.67; 5.75) p=0.22	2.27 (0.98; 5.27) p=0.056	1.18 (0.40; 3.50) p=0.76	1.08 (0.37; 3.17) p=0.89	
	1 week - 1 month vs. >1 year	0.74 (0.13; 4.39) p=0.74	2.19 (0.94; 5.12) p=0.070	2.67 (1.13; 6.31) p=0.026	1.12 (0.36; 3.42) p=0.85	
	1 month - 6 months vs. >1 year	1.39 (0.47; 4.07) p=0.55	1.52 (0.66; 3.54) p=0.33	1.21 (0.47; 3.08) p=0.69	2.19 (0.99; 4.84) p=0.052	
	6 months - 1 year vs. >1 year	1.36 (0.37; 5.01) p=0.64	1.90 (0.77; 4.69) p=0.16	1.17 (0.38; 3.59) p=0.78	2.48 (1.00; 6.13) p=0.049	
Family member's age per 10-year age category		0.91 (0.63; 1.31) p=0.61	1.30 (1.00; 1.68) p=0.048	1.32 (1.00; 1.75) p=0.051	1.67 (1.24; 2.24) p=0.0007	p=0.003
Family member's sex	Female vs. Male	1.27 (0.54; 2.99) p=0.58	3.80 (1.66; 8.72) p=0.002	1.44 (0.73; 2.84) p=0.29	1.27 (0.65; 2.47) p=0.48	p=0.040
Family member's country of birth	Outside Sweden vs. Sweden	1.25 (0.36; 4.30) p=0.72	0.55 (0.16; 1.89) p=0.34	1.57 (0.60; 4.09) p=0.35	0.48 (0.12; 1.96) p=0.30	p=0.47
Family member's parents' country of birth	Outside Sweden vs. Sweden	1.12 (0.38; 3.31) p=0.84	1.09 (0.49; 2.43) p=0.83	1.76 (0.79; 3.90) p=0.17	0.38 (0.10; 1.43) p=0.15	p=0.29
Family member's educational level	Higher secondary vs. Elementary-secondary	1.04 (0.38; 2.83) p=0.94	1.56 (0.70; 3.51) p=0.28	0.69 (0.30; 1.58) p=0.38	0.29 (0.13; 0.64) p=0.002	p=0.0009
	Higher vs. Elementary-secondary	0.77 (0.29; 2.10) p=0.61	1.79 (0.82; 3.88) p=0.14	0.81 (0.39; 1.70) p=0.58	0.19 (0.08; 0.43) p<0.0001	
Family member's occupation	Employed/ Student** vs. Unemployed/ Pensioner***	0.87 (0.39; 1.92) p=0.73	0.70 (0.40; 1.24) p=0.23	0.47 (0.24; 0.90) p=0.024	0.49 (0.26; 0.94) p=0.032	p=0.081

		Odds Ratio (95% CI) vs at home				
Variable	Comparison	Family or friend's home	Hospice	Hospital	Nursing home	Global test of association†
Support for family members*	Did not need support vs. Yes	2.45 (0.72; 8.31) p=0.15	2.68 (1.04; 6.90) p=0.040	1.29 (0.40; 4.13) p=0.67	1.97 (0.65; 5.97) p=0.23	p=0.89
	No, did not try to get more vs. Yes	1.40 (0.38; 5.14) p=0.62	0.78 (0.21; 2.91) p=0.71	0.50 (0.11; 2.25) p=0.37	0.99 (0.30; 3.25) p=0.99	
	No, tried to get more vs. Yes	1.83 (0.55; 6.10) p=0.33	1.36 (0.46; 3.99) p=0.58	0.33 (0.04; 2.62) p=0.30	1.22 (0.40; 3.73) p=0.73	
	Yes, some vs. Yes	0.78 (0.26; 2.32) p=0.65	1.13 (0.56; 2.27) p=0.73	0.80 (0.37; 1.75) p=0.58	0.83 (0.37; 1.84) p=0.64	
Quality of care	Poor/Fair vs. Good	1.42 (0.46; 4.40) p=0.54	0.78 (0.33; 1.82) p=0.56	0.70 (0.26; 1.89) p=0.48	0.51 (0.19; 1.38) p=0.19	p=0.88
	Excellent/ Outstanding vs. Good	1.59 (0.62; 4.13) p=0.34	0.89 (0.47; 1.69) p=0.72	1.02 (0.51; 2.06) p=0.95	0.83 (0.44; 1.56) p=0.57	
Ill person participation in decision-making	As much as wanted or more vs. Liked to be more involved	1.00 (0.32; 3.10) p=1.00	1.21 (0.51; 2.86) p=0.66	1.10 (0.44; 2.80) p=0.83	1.89 (0.64; 5.57) p=0.25	p=0.83
Family participation in decision-making	As much as wanted or more vs. Liked to be more involved	1.05 (0.44; 2.53) p=0.91	1.04 (0.54; 1.99) p=0.91	1.17 (0.57; 2.42) p=0.66	0.78 (0.38; 1.59) p=0.49	p=0.92
Ill person's preferred place of death	Hospital/Hospice/Nursing home vs. At home	1.07 (0.20; 5.90) p=0.93	2.03 (0.69; 5.93) p=0.20	1.09 (0.32; 3.68) p=0.89	0.91 (0.16; 5.08) p=0.91	p=0.74
	Did not matter vs. At home	1.77 (0.36; 8.76) p=0.48	1.05 (0.27; 4.16) p=0.94	0.56 (0.09; 3.43) p=0.53	1.97 (0.57; 6.83) p=0.28	
Death in the right place	Yes vs. No	0.41 (0.14; 1.25) p=0.12	0.71 (0.26; 1.96) p=0.51	1.19 (0.36; 3.99) p=0.78	0.56 (0.22; 1.47) p=0.24	p=0.46
Relation to deceased person	Partner vs. Child	0.92 (0.35; 2.39) p=0.86	2.17 (1.16; 4.08) p=0.016	3.05 (1.52; 6.10) p=0.002	3.30 (1.70; 6.38) p=0.0004	p=0.006
	Other vs. Child	1.13 (0.33; 3.95) p=0.84	2.01 (0.91; 4.43) p=0.085	2.06 (0.78; 5.44) p=0.14	1.74 (0.65; 4.67) p=0.27	
RAND-12 physical component score per 10 units		0.83 (0.57; 1.21) p=0.34	0.98 (0.72; 1.35) p=0.92	0.72 (0.54; 0.97) p=0.030	0.80 (0.61; 1.06) p=0.13	p=0.20
RAND-12 mental component score per 10 units		0.92 (0.66; 1.28) p=0.63	0.84 (0.67; 1.06) p=0.14	0.80 (0.62; 1.02) p=0.077	1.02 (0.78; 1.33) p=0.89	p=0.34

* One pseudo-count was added to each combination of levels of explanatory variables and response variable to avoid complete or quasi-complete separation of data points.

** Employed/Self-employed/Homemaker/Student.

*** Unemployed/Pensioner/Long-term sick/Other.

† Multivariate likelihood ratio test of association with preferred place of care.

Supplementary table 2. Where would you most like to die? Results from univariable multinomial logistic regression analysis. Odds Ratio (95% CI) vs. At home.

		Odds Ratio (95% CI) vs At home				
Variable	Comparison	Family or friends home	Hospice	Hospital	Nursing home	Global test of association†
Deceased person's age per 10 years		1.00 (0.65; 1.54) p=1.00	1.14 (0.90; 1.43) p=0.28	1.00 (0.76; 1.32) p=1.00	0.92 (0.69; 1.23) p=0.57	p=0.77
Deceased person's sex	Female vs. Male	1.34 (0.50; 3.65) p=0.56	0.60 (0.37; 0.97) p=0.038	0.70 (0.39; 1.26) p=0.23	0.54 (0.28; 1.04) p=0.065	p=0.13
Cause of death	Cancer vs. Non-cancer	1.63 (0.65; 4.14) p=0.30	1.06 (0.64; 1.77) p=0.82	0.84 (0.44; 1.62) p=0.60	1.18 (0.58; 2.38) p=0.65	p=0.77
Time of illness before death*	<24 h vs. >1 year	0.65 (0.13; 3.14) p=0.59	0.52 (0.20; 1.36) p=0.18	0.83 (0.29; 2.39) p=0.73	0.45 (0.10; 2.02) p=0.30	p=0.99
	24 h - 1 week vs. >1 year	1.49 (0.43; 5.15) p=0.53	1.58 (0.75; 3.36) p=0.23	1.29 (0.48; 3.46) p=0.61	1.21 (0.44; 3.35) p=0.71	
	1 week - 1 month vs. >1 year	0.87 (0.19; 3.94) p=0.86	1.08 (0.51; 2.27) p=0.84	1.69 (0.72; 3.93) p=0.23	0.85 (0.28; 2.57) p=0.78	
	1 month - 6 months vs. >1 year	0.98 (0.27; 3.60) p=0.98	0.87 (0.41; 1.83) p=0.72	1.25 (0.54; 2.89) p=0.60	1.29 (0.54; 3.08) p=0.57	
	6 months - 1 year vs. >1 year	0.52 (0.07; 3.64) p=0.51	0.91 (0.41; 2.01) p=0.81	1.17 (0.45; 3.04) p=0.74	0.88 (0.29; 2.67) p=0.83	
Family member's age per 10-year age category		1.10 (0.73; 1.66) p=0.65	1.26 (1.02; 1.55) p=0.032	1.22 (0.95; 1.57) p=0.12	1.77 (1.28; 2.44) p=0.0006	p=0.007
Family member's sex	Female vs. Male	0.60 (0.23; 1.60) p=0.31	2.43 (1.35; 4.39) p=0.003	1.16 (0.62; 2.20) p=0.64	1.14 (0.56; 2.32) p=0.72	p=0.038
Family member's country of birth	Outside Sweden vs. Sweden	1.37 (0.30; 6.28) p=0.69	0.46 (0.15; 1.40) p=0.17	1.45 (0.59; 3.54) p=0.41	0.30 (0.03; 2.62) p=0.28	p=0.33
Family member's parents' country of birth	Outside Sweden vs. Sweden	1.00 (0.24; 4.16) p=1.00	1.09 (0.54; 2.21) p=0.80	1.34 (0.62; 2.91) p=0.46	0.41 (0.10; 1.67) p=0.21	p=0.62

		Odds Ratio (95% CI) vs At home				
Variable	Comparison	Family or friends home	Hospice	Hospital	Nursing home	Global test of association†
Family member’s educational level	Higher secondary vs. Elementary-secondary	0.42 (0.12; 1.51) p=0.18	1.00 (0.51; 1.95) p=0.99	0.82 (0.39; 1.73) p=0.61	0.27 (0.12; 0.65) p=0.003	p=0.002
	Higher vs. Elementary-secondary	0.73 (0.25; 2.11) p=0.56	1.65 (0.88; 3.09) p=0.12	0.84 (0.40; 1.77) p=0.64	0.21 (0.09; 0.52) p=0.0008	
Family member's occupation	Employed/Student** vs. Unemployed/Pensioner***	0.50 (0.19; 1.32) p=0.16	0.71 (0.44; 1.16) p=0.17	0.52 (0.28; 0.94) p=0.030	0.29 (0.13; 0.64) p=0.002	p=0.011
Support for family members*	Did not need support vs. Yes	1.99 (0.50; 7.91) p=0.33	1.05 (0.44; 2.48) p=0.91	0.78 (0.27; 2.21) p=0.63	0.46 (0.10; 2.15) p=0.33	p=0.42
	No, did not try to get more vs. Yes	1.44 (0.29; 7.14) p=0.65	0.61 (0.21; 1.77) p=0.36	0.63 (0.17; 2.29) p=0.48	0.94 (0.30; 2.95) p=0.91	
	No, tried to get more vs. Yes	1.97 (0.49; 7.97) p=0.34	0.83 (0.34; 2.05) p=0.69	0.16 (0.02; 1.31) p=0.088	0.28 (0.04; 1.88) p=0.19	
	Yes, some vs. Yes	1.13 (0.36; 3.55) p=0.84	0.68 (0.37; 1.26) p=0.22	0.38 (0.17; 0.87) p=0.022	0.35 (0.14; 0.91) p=0.032	
Quality of care	Poor/Fair vs. Good	2.55 (0.62; 10.56) p=0.20	0.73 (0.36; 1.49) p=0.39	0.74 (0.32; 1.73) p=0.49	0.26 (0.05; 1.37) p=0.11	p=0.28
	Excellent/Outstanding vs. Good	2.68 (0.75; 9.58) p=0.13	1.31 (0.76; 2.28) p=0.34	1.29 (0.68; 2.46) p=0.43	1.39 (0.69; 2.78) p=0.36	
Ill person participation in decision-making	As much as wanted or more vs. Liked to be more involved	0.83 (0.20; 3.39) p=0.79	0.94 (0.49; 1.81) p=0.85	1.97 (0.60; 6.44) p=0.26	1.57 (0.48; 5.10) p=0.45	p=0.69
Family participation in decision-making	As much as wanted or more vs. Liked to be more involved	0.77 (0.27; 2.14) p=0.61	1.37 (0.77; 2.47) p=0.29	1.15 (0.58; 2.25) p=0.69	0.83 (0.39; 1.80) p=0.64	p=0.71
Ill person's preferred place of death*	Hospital/Hospice/Nursing home vs. At home	0.61 (0.05; 6.75) p=0.68	2.15 (0.82; 5.64) p=0.12	1.50 (0.47; 4.83) p=0.49	0.54 (0.11; 2.69) p=0.45	p=0.57
	Did not matter vs. At home	2.08 (0.37; 11.76) p=0.40	1.10 (0.36; 3.31) p=0.87	0.99 (0.28; 3.53) p=0.99	0.78 (0.27; 2.27) p=0.64	

		Odds Ratio (95% CI) vs At home				
Variable	Comparison	Family or friends home	Hospice	Hospital	Nursing home	Global test of association†
**Death in the right place***	Yes vs. No	0.44 (0.15; 1.28) p=0.13	1.20 (0.54; 2.65) p=0.65	2.95 (0.85; 10.20) p=0.088	2.97 (0.48; 18.40) p=0.24	p=0.12
**Relation to deceased person**	Partner vs. Child	2.41 (0.85; 6.85) p=0.098	1.79 (1.03; 3.10) p=0.038	2.14 (1.14; 4.02) p=0.018	6.58 (3.00; 14.46) p<0.0001	p=0.001
	Other vs. Child	1.80 (0.41; 7.94) p=0.44	1.56 (0.78; 3.09) p=0.21	0.68 (0.23; 2.04) p=0.49	3.40 (1.22; 9.48) p=0.019	
**RAND-12 physical component score per 10 units**		0.92 (0.55; 1.54) p=0.75	0.83 (0.64; 1.08) p=0.16	0.75 (0.55; 1.02) p=0.067	0.75 (0.55; 1.03) p=0.075	p=0.28
**RAND-12 mental component score per 10 units**		0.98 (0.66; 1.47) p=0.93	1.08 (0.87; 1.33) p=0.51	1.03 (0.80; 1.34) p=0.79	0.94 (0.72; 1.25) p=0.69	p=0.94

* One pseudo-count was added to each combination of levels of explanatory variables and response variable to avoid complete or quasi-complete separation of data points.

** Employed/Self-employed/Homemaker/Student.

*** Unemployed/Pensioner/Long-term sick/Other.

† Multivariate likelihood ratio test of association with preferred place of death.
